# The Trifecta of Industry, Academic, and Health System Partnership to Improve Mental Health Care Through Smartphone-Based Remote Patient Monitoring: Development and Usability Study

**DOI:** 10.2196/57624

**Published:** 2025-01-07

**Authors:** C Neill Epperson, Rachel Davis, Allison Dempsey, Heinrich C Haller, David J Kupfer, Tiffany Love, Pamela M Villarreal, Mark Matthews, Susan L Moore, Kimberly Muller, Christopher D Schneck, Jessica L Scott, Richard D Zane, Ellen Frank

**Affiliations:** 1 Department of Psychiatry School of Medicine University of Colorado Anschutz Medical Campus Aurora, CO United States; 2 Department of Psychiatry School of Medicine University of Pittsburgh Pittsburgh, PA United States; 3 UCHealth CARE Innovations Center Anschutz Medical Campus University of Colorado Health System Aurora, CO United States; 4 Health Rhythms New York, NY United States; 5 CU Innovations University of Colorado Anschutz Medical Campus Aurora, CO United States; 6 Department of Emergency Medicine School of Medicine University of Colorado Anschutz Medical Campus Aurora, CO United States

**Keywords:** digital health, mobile intervention, telepsychiatry, artificial intelligence, psychiatry, mental health, depression, mood, bipolar, monitor, diagnostic tool, diagnosis, electronic health record, EHR, alert, notification, prediction, mHealth, mobile health, smartphone, passive, self-reported, patient generated

## Abstract

**Background:**

Mental health treatment is hindered by the limited number of mental health care providers and the infrequency of care. Digital mental health technology can help supplement treatment by remotely monitoring patient symptoms and predicting mental health crises in between clinical visits. However, the feasibility of digital mental health technologies has not yet been sufficiently explored. Rhythms, from the company Health Rhythms, is a smartphone platform that uses passively acquired smartphone data with artificial intelligence and predictive analytics to alert patients and providers to an emerging mental health crisis.

**Objective:**

The objective of this study was to test the feasibility and acceptability of Rhythms among patients attending an academic psychiatric outpatient clinic.

**Methods:**

Our group embedded Rhythms into the electronic health record of a large health system. Patients with a diagnosis of major depressive disorder, bipolar disorder, or other mood disorder were contacted online and enrolled for a 6-week trial of Rhythms. Participants provided data by completing electronic surveys as well as by active and passive use of Rhythms. Emergent and urgent alerts were monitored and managed according to passively collected data and patient self-ratings. A purposively sampled group of participants also participated in qualitative interviews about their experience with Rhythms at the end of the study.

**Results:**

Of the 104 participants, 89 (85.6%) completed 6 weeks of monitoring. The majority of the participants were women (72/104, 69.2%), White (84/104, 80.8%), and non-Hispanic (100/104, 96.2%) and had a diagnosis of major depressive disorder (71/104, 68.3%). Two emergent alerts and 19 urgent alerts were received and managed according to protocol over 16 weeks. More than two-thirds (63/87, 72%) of those participating continued to use Rhythms after study completion. Comments from participants indicated appreciation for greater self-awareness and provider connection, while providers reported that Rhythms provided a more nuanced understanding of patient experience between clinical visits.

**Conclusions:**

Rhythms is a user-friendly, electronic health record–adaptable, smartphone-based tool that provides patients and providers with a greater understanding of patient mental health status. Integration of Rhythms into health systems has the potential to facilitate mental health care and improve the experience of both patients and providers.

## Introduction

Mental disorders are predicted to be the leading cause of disease burden worldwide by 2030 [1]. However, there is a tremendous mismatch between the number of psychiatrists, psychologists, and other mental health care providers and individuals in need of treatment for mental illness, including substance use disorders. In the United States alone, 160 million people reside where there is a shortage of mental health professionals [2]. Roughly 56% of counties are without a practicing psychiatrist, while 33% of counties are without a licensed psychologist [3]. The workforce is expected to be short 38,821 psychiatrists by 2024 [4]. In the United States, the majority of individuals seeking treatment for depression, anxiety, or any mental illness receive care from their primary care physician (PCPs), and PCPs provide almost a third of the care for serious mental illness [5].

On average, a PCP addresses between 3 to 5 problems, including psychiatric problems, during a typical encounter [6,7]. Furthermore, the majority of PCPs cannot see patients as frequently as may be necessary to adequately monitor a patients’ mental illness and response to treatment, nor do they have a mental health specialist to whom they can refer their more concerning patients [8]. In the context of the current mental health landscape, remote monitoring of patient mental well-being between visits is poised to play a major role in the redesigning of mental health care, similar to what is occurring with remote management of myriad other conditions [9,10].

Ideally, remotely acquired data would be integrated into the electronic health record (EHR) and readily available to the patient’s provider for interpretation and intervention if needed. With remote patient monitoring, the documentation of history since the previous appointment is quicker, the challenges of patient recall with respect to their signs and symptoms are obviated, and there is a foundation for patients to participate to a greater degree in their own psychiatric care by tracking their symptoms alongside their provider.

Clinical, patient-generated, app-acquired health data that are embedded and presented into the EHR workflow provide the opportunity for interprofessional use. This is particularly important when patients have multiple comorbid medical conditions that impact brain health and vice versa. Furthermore, patient engagement with health apps, including those that are mental health related, declines dramatically over the first few weeks of use, in some cases dropping below 10% within 2 weeks [11,12]. An ideal app may rely less upon active user engagement for data collection and to a greater degree on passively acquired data that reduce patient burden, which then trigger the completion of symptom self-reports using nationally accepted standardized ratings when passively acquired patient-generated health data (PGHD) indicate a relapse is about to occur.

To address these deficits in the digital mental health space, an academic-industry collaborative team was formed to test the feasibility and acceptability of industry start-up Health Rhythms’ smartphone solution called Rhythms for use across a large health system. Previous research has demonstrated that Rhythms’ proprietary artificial intelligence (AI) algorithm applied to passively collected data indicative of behavioral vital signs (eg, sleep patterns, activity measures, and sociability) can predict a relapse of depression 7 days before the patient’s self-report of relapse with 90% accuracy [13-15]. This collaborative team reports the results of the EHR integration, results of the feasibility testing of the urgent and emergent alert algorithms, effectiveness of the 24/7 virtual patient monitoring system, user acceptability in terms of both patient use of and satisfaction with Rhythms, and provider impressions of the impact of Rhythms on patient care.

## Methods

### Collaborative Team

Health Rhythms is a technology company that was founded by experienced researchers and clinicians (EF and DK) in the assessment, treatment, and research of mental illness, particularly bipolar disorder and depression, in partnership with international leaders (MM) in behavioral sensing technology focused on mental health. The University of Colorado (CU) Anschutz Medical Campus Department of Psychiatry is a large department (500+ faculty) with a strong commitment to the incorporation of digital health and technology in the care of patients with serious mental illness. CU Innovations brings together industry partners, entrepreneurs, and investors to help CU researchers create biomedical technology that improves care delivery, health outcomes, and patient quality of life worldwide. University of Colorado Health (UCHealth) is a not-for-profit health care system across Colorado, southern Wyoming, and western Nebraska with an academic medical center, the University of Colorado Hospital (UCH), that is affiliated with the CU School of Medicine. The CARE Innovation Center at UCHealth partners with CU to create a comprehensive platform of resources both to conduct clinical and biomedical research and to transform real-world clinical practice together with industry partners or faculty entrepreneurs.

### App Development

The Rhythms app was developed using a participatory design process [16], an approach that seeks to deeply involve end users in the design process. A weekly meeting between the Health Rhythms team and the UCHealth clinical team was the primary means to engage in this process. It focused on understanding end-user needs, existing clinical workflows, and seeking feedback on early prototypes.

A key decision resulting from this process was to integrate the Health Rhythms software development kit (SDK) into UCHealth’s MyChart mobile app, rather than providing a distinct separate app that participants would have to download. Health Rhythms’ SDK is a modular piece of software that can be integrated easily into native iOS or Android apps. Although this decision required significant technical work, the team anticipated that it would significantly streamline the patient end-user experience, since they would only have to install and use 1 app, and would help maintain user privacy and security.

Once integrated into the UCHealth MyChart app and end users have provided explicit permissions during onboarding, the SDK collects data continuously in the background from the activity, location, and display smartphone sensors. These data can then be used to automatically assess behavior patterns derived from these data streams including how much time a person is spending at home, levels of physical activity, estimates of sleep timing, and duration and interaction patterns with the smartphone itself.

Safeguarding patient privacy was paramount. Users were uniquely identified using a nonidentifiable identifier that was passed to Health Rhythms. The Rhythms app did not collect any identifiable data that falls under the protections of the Health Insurance Portability and Accountability Act (HIPAA) such as birth dates, home addresses, or social security numbers, and the SDK did not collect any data from texts, calls, photos, internet browsing, or other content. All data, including GPS location, are encrypted using state of the art encryption (ie, a 256-bit key) on the device, in transmission and in the cloud. The sensor data for each patient were computed into daily nonidentifiable behavioral summaries that were then transmitted into the EHR using standard Fast Healthcare Interoperability Resources end points. No unprocessed sensor data, including GPS, were transferred into the EHR.

While there is always a risk of data breach in such systems, the following measures were implemented to mitigate this risk. The team applied the principle of data minimization, reducing the collection, storage, and use of personal data to what is strictly necessary to assess individual behavioral health. Patient end users had the agency to rescind permissions for sensor data collection at any time by a “Manage Settings” screen, and transparent, easy-to-understand messaging during onboarding communicated clearly what data were collected and how they would be used. Finally, Health Rhythms maintained General Data Protection Regulation and California Consumer Privacy Act certification and conducted regular security tests by professional third parties.

### AI Methods

The Rhythms system collects passive sensing data, comprising location, pedometer, activity, and device data, from the onboard sensors in smartphones. Each day, these data are processed into 65 behavioral inferences for each person. These inferences encompass measures related to physical and social activity, stability or instability of the person’s weekly routine, sleep, and smartphone use. For example, time at home is an inference derived from location data about how much time a person is spending at home.

These behavioral inferences are used in 2 ways. First, heuristics related to these inferences are used to trigger a request for a self-report from participants. A total of 15 static heuristics, empirically derived from previous datasets, dynamically prompted individuals to complete self-reported assessments. For example, if an individual had a diagnosis of major depression and spent a long time at home for over 7 days, then a self-report was triggered. Second, the behavioral inferences were fed into a logistic regression machine learning model that provided a continuous and passive estimate of a person’s risk for a relapse in depression, defined as scoring greater than 10 on the Patient Health Questionnaire 9-Item (PHQ-9). This model aims to be predictive of how a given person would answer specific questions on the PHQ-9. Model outputs with a high probability for a high PHQ-9 score were used to flag individuals as a medium alert on the dashboard if there was no corroborating self-report.

### Participant Criteria and Recruitment

Potential participants were residents of Colorado; aged between 18 and 89 years; were the primary user of a mobile phone that supported iOS12 or above; enrolled in MyHealthConnection (MHC), which is UCH’s EHR patient portal; and were willing to enroll their device into Rhythms.

The UCH’s EHR (EPIC) was used to search the caseload of participating clinicians in the CU Department of Psychiatry to identify patients who carry the diagnosis of depressive disorder including major depressive disorder (MDD), dysthymia, depressive disorder due to another medical condition, or bipolar disorder. Providers reviewed the list to confirm that individuals were current patients and would be clinically appropriate for contact about Rhythms.

Recruitment was conducted virtually. Patients were contacted initially through MHC with a general message describing the study. Due to poor initial response to the MHC outreach, a personalized email letter was sent from their provider. Potential participants were contacted up to 3 times by a study coordinator, and screening appointments were scheduled if they expressed interest in the study. After consenting and screening, the study coordinator reviewed how to download the app and interact with the platform and placed an order for Rhythms in EPIC. Participants were considered lost to follow-up if they did not respond to 3 contacts by the study team after enrollment.

**Figure 1:**
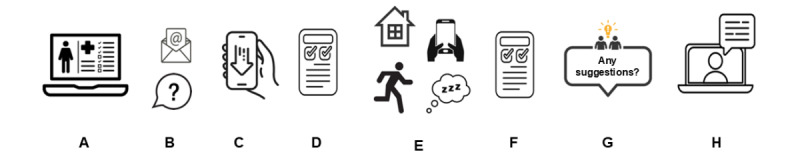
Study flow chart: (A) potential participants identified using the electronic health record in collaboration with providers; (B) potential participants contacted up to three times; (C) participants (who met criteria and signed consent) downloaded Rhythms and were instructed in its use; (D) participants completed baseline assessments; (E) participants continued their daily activities, with Rhythms passively collecting data for 6 weeks; (F) participants completed baseline assessment again at end of study; (G) participants completed a survey of likes and dislikes with respect to Rhythms; and (H) selected group underwent qualitative interviews about Rhythms use.

Throughout the study, participants provided data by completing electronic surveys sent by the study team (baseline, end of study [6 weeks], and when triggered by algorithm), as well as by active and passive use of the Rhythms platform. A purposively sampled [17,18] group of participants participated in qualitative interviews with a member of the study team at the end of the study. An overview of the study design and procedures is presented in Figure 1.

### Data Collected by Rhythms

#### Passively Measured Outcomes

Rhythms acquired proxy information regarding sleep quality, activity level, and sociability. The AI algorithm uses passively collected data to detect deviations from the individual’s baseline and triggers a push to complete the self-report ratings described below. During the course of this study, the participant could not view their own passively acquired data.

#### Self-Report Measures

Per study protocol, self-report measures were administered at baseline and end of study. In addition, self-report measures were pushed by Rhythms to the participant during the study period when the AI algorithm detected a deviation in passively collected data indicative of worsening of symptoms of depression, anxiety, or mania. Participant self-ratings included the following:

#### PHQ-9 Instrument

The PHQ-9 asks the individual to rate the severity of symptoms over the past 2 weeks on a 4-point scale ranging from “not at all” to “nearly every day.” Endorsement of either depressed mood or anhedonia (PHQ-2) triggers further evaluation with the PHQ-9, which assesses for the presence of other criteria for MDD. In multiple studies, PHQ-9 scores >10 had a sensitivity of 88% and a specificity of 88% for MDD [19-21].

#### Columbia-Suicide Symptom Severity Rating Scale

The Columbia-Suicide Symptom Severity Rating Scale (C-SSRS) is used to assess severity and immediacy of suicide risk using four constructs: (1) severity of suicidal ideation, (2) intensity of ideation, (3) behavior, and (4) lethality. The instrument has been reported to have a 67% sensitivity and 76% specificity for identifying suicidal behaviors [22,23].

#### Generalized Anxiety Disorder 7-Item

The Generalized Anxiety Disorder 7-Item (GAD-7) is used to measure the severity of generalized anxiety disorder symptoms by asking individuals to rate the severity of symptoms on a 4-point scale with ranging from “not at all” to “nearly every day.” Scores of 5, 10, and 15 represent cutoff points for mild, moderate, and severe anxiety, respectively [24].

#### Altman Self-Rating Mania Scale

The Altman Self-Rating Mania Scale (ASRM) is a 5-item, self-reported scale to assess positive mood, self-confidence, sleep patterns, speech patterns and amount, and motor activity, which are rated on a scale of “0” or “normal” to “4” or “overtly manic.” Total scores range from 0 to 20, with scores equal to or higher than 6 indicating a higher likelihood of manic or hypomanic symptoms [25].

### Participant-Reported Measures Collected by Study Team

#### Postintervention Feasibility and Satisfaction Survey

At study end, participants completed a survey about what they liked or disliked most about Rhythms, their interest in continued use, challenges or barriers to use, and any concerns that they may have had regarding alerting and monitoring through the use of Rhythms.

#### Qualitative Interviews

The qualitative in-depth interviews were conducted by Zoom (Zoom Video Communications) using a semistructured interview guide to ensure key topics were discussed while also allowing for emergence and exploration of topics important to the participants. The main interview questions for patients focused on their perceived benefits from and drawbacks of the Rhythms’ system and suggestions for improving Rhythms’ features and use.

Providers also completed a feedback survey at study end. Main questions for providers included their experience integrating the Rhythms system into their workflow, how the system was perceived to impact the quality of care, and the efficiency of providing personalized treatment for patients.

### Determining Alert Structure

Based upon previous research with Rhythms [14,15,17,26] and consensus of subject matter experts in the treatment of MDD, anxiety, and bipolar disorder, Rhythms was programmed to send urgent or emergent alerts to UCHealth’s Virtual Behavioral Health Center (VBHC) or the Department of Psychiatry’s care coordinator based upon passively acquired data and participant self-ratings (Textbox 1).

**Urgent and emergent alerts.**

**Urgent alert**

- Physician Health Questionnaire-9 item score is 20-27, when the previous score was lower.
- Columbia-Suicide Severity Rating Scale question 3=yes.
- Altman Self-Rating for Mania Scale score is ≥11.
- Altman Self-Rating for Mania Scale score is <11 and score for question 3=3 or 4.
- If bipolar disorder diagnosis and Rhythms detect a 40% decrease in sleep.
- If bipolar disorder diagnosis and Rhythms monitoring shows no sleep in 2 days.
- Generalized Anxiety Disorder-7 item score ranges from 15-21, when the previous score was not in this range.

**Emergent alert**

- Columbia-Suicide Severity Rating Scale questions 4, 5, or 6=yes.
- Altman Self-Rating for Mania Scale total score >11 and answer to question 3=4.

The following specific self-rating questions pertained to the alert structure: C-SSRS question 3: Active suicidal ideation with any methods (not plan) without intent to act; C-SSRS question 4: Active suicidal ideation with some intent to act, without specific plan; C-SSRS question 5: Active suicidal ideation with specific plan and intent; C-SSRS question 6: Have you ever done anything, started to do anything, or prepared to do anything to end your life?; and ASRM question 3: Regarding Sleep Patterns: score of 3=“I frequently need less sleep than usual.” and score of 4=“I can go all day and night without any sleep and not feel tired*.*”

Per protocol, a care coordinator from the VBHC or the Department of Psychiatry called the participant up to 3 times within 24 hours in response to an *urgent* alert. The VBHC monitor covered alerts over weekends, holidays, and after hours, while the care coordinator supporting the participant’s primary mental health care provider covered during regular work hours. When the participant was reached by the VBHC, a brief safety assessment was conducted; participants were offered the chance to speak to an on-call clinician for the VBHC and referred to their current provider if appropriate. In the case of clinic care coordinators, the participant was assessed with respect to safety, possible medication changes, and need for earlier appointment with their provider.

In response to *emergent* alerts, Health Rhythms sent a page to the VBHC monitor. The pager beeped every 5 minutes until the alert was acknowledged by the monitor in the EHR. All emergent alerts were managed by the VBHC monitor with the expectation that a call to the patient would be initiated within 15 minutes of the alert. Upon reaching the patient, a safety assessment was conducted, and an appropriate disposition was managed. If after 3 attempts there was no contact with the patient, the VBHC monitor called emergency services to conduct a welfare check at the address listed for the participant.

Initially, participants received a message each time they completed self-report measures that they could receive a call from the VBHC based upon how they answered the questionnaires. This caused confusion for participants and the process was changed such that only participants who had triggered an emergent or urgent alert would get the message that they would receive a call.

### Data Analysis

Demographics and baseline behavioral rating scores as well as number and nature of alerts were analyzed descriptively with means (SDs) and frequencies (percentages). Postintervention survey data were summarized descriptively for fixed-choice items. Open-ended survey items and interview transcripts were analyzed qualitatively. Human-adjudicated transcription of interview recordings was performed with Otter.ai, a proprietary AI-powered note taking engine, to generate initial transcripts, after which a member of the study team reviewed the transcripts while simultaneously listening to the recordings to ensure there were no errors in transcription.

A rapid qualitative analysis approach was used for content analysis of open-ended survey and interview transcript data. We first deductively explored and identified common themes that appeared in participants’ responses. Two members of the research team separately reviewed the data and identified keywords and topics that are relevant to our research questions (eg, most liked and disliked features of the Rhythms’ system, facilitators and barriers to using the Rhythms system, and suggestions to improve the system). During this process, each reviewer independently summarized how frequently common items were reported by study participants. The reviewers compared their results, discussed and resolved any discrepancies, reached consensus on the latent themes that had emerged in analysis, and generated a summary.

### Ethical Considerations

This project was approved by the University of Colorado Multiple Institution Review Board (COMIRB; 21-4903), and participants gave electronic informed consent. Participants received US $25 in compensation for completing the screening, enrollment, and baseline surveys, and an additional US $50 for completing the postintervention surveys as described below. All data were deidentified for analysis and presented anonymously.

## Results

### Participants

Of 394 patients who were informed about the study through MHC or email, 178 (45.2%) did not respond, 26 (6.7%) were contacted but lost to follow-up before enrollment, 86 (21.8%) were screened but did not enroll, and 104 (26.4%) were enrolled within 8 weeks of study initiation. The most common reason (60/86, 69.8%) for not meeting study criteria was having a phone using the Android operating system.

The study sample comprised 104 individuals; the majority identified as women (n=72 69.2%), non-Hispanic (n=100 96.2%), and White (n=84 80.8%). The average age was 42.1 (SD 15.9) years. The majority of participants had MDD (n=71 68.3%), while 23 (22.2%) individuals had either bipolar I or II disorder and 10 individuals (9.6%) had “other mood disorder” (Table 1).

Between enrollment and study completion, 17 (16.3%) of the 104 enrolled participants were lost to follow-up, withdrew from the study, or did not complete the final survey. Of note, among the 87 (83.7%) completers out of 104, a total of 72% (63/87) continued using Rhythms despite no longer being compensated for doing so.

**Table 1:** Participant characteristics and baseline behavioral ratings.

Variable		Value (n=104)
**Age (years), mean (SD)**		42.1 (15.9)
**Gender, n (%)**		
	Women	72 (69.2)
	Men	29 (27.9)
	Transgender or nonbinary	3 (2.9)
**Ethnicity, n (%)**		
	Hispanic	3 (2.9)
	Non-Hispanic	100 (96.2)
	Not answered	1 (1)
**Race, n (%)**		
	Asian	9 (8.6)
	Black or African American	1 (1)
	American Indian or Alaska Native	1 (1)
	White	84 (80.8)
	More than one race	4 (3.8)
	Other or prefer not to answer	5 (4.8)
**Diagnosis, n (%)**		
	Major depressive disorder	71 (68.3)
	Bipolar 1 disorder	12 (11.5)
	Bipolar II disorder	11 (10.6)
	Other mood disorder	10 (9.6)
**Ratings, mean (SD)**		
	Physician Health Questionnaire 9-Item	6.5 (5.3)
	Generalized Anxiety Disorder 7-Item	5.9 (4.8)
	Altman Self-Rating Mania Scale	2.2 (2.7)

### Urgent and Emergent Alerts

**Figure 2:**
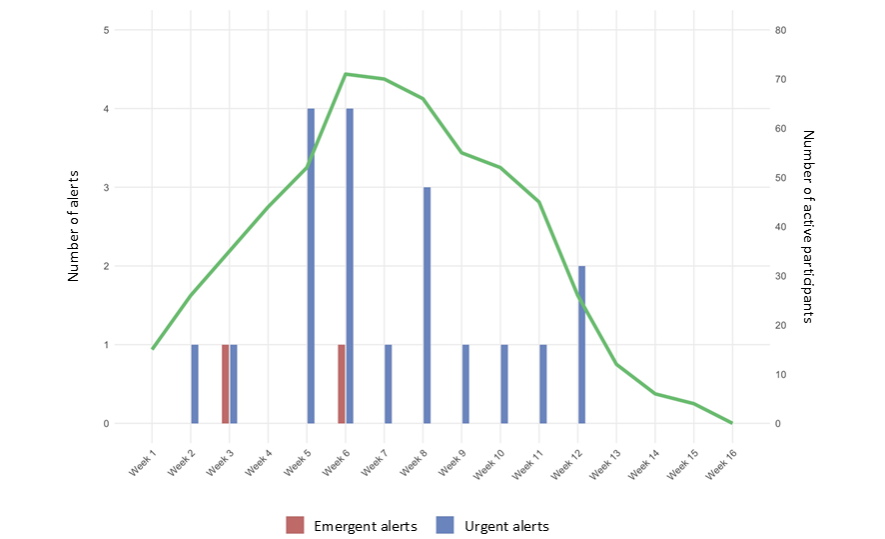
Urgent and emergent alerts across time and by number of active participants.

Over the course of the entire 16-week study period, the monitoring of 104 individuals with Rhythms for 6 weeks each resulted in 2 emergent alerts and 19 urgent alerts. The largest number of participants being monitored at 1 time was 92 and the average was 64. The greatest number of alerts on any given day was 3 (all urgent, 1 day only) when there were 58 participants being monitored (Figure 2).

The first emergent alert was triggered by a participant answering ASRM question 3 about sleep with a “4,” indicating “I can go all day and night without any sleep and not feel tired.” The second emergent alert was triggered by the participant answering yes to C-SSRS question 6, indicating that they “had ever done anything, started to do anything or prepared to do anything to end their life.” The primary (n=15) trigger for urgent alerts was answering “yes” to question 3 on the C-SSRS, indicating that the participant had active suicidal ideation without method or intent to act. The second most common (n=12) reason for urgent alerts was a GAD-7 score indicating severe anxiety. Three urgent alerts occurred because of the ASRM indicating mania or hypomania based upon total ASRM or response to question 3 regarding need for sleep.

All calls to participants were managed within the specified alert time. Eleven (58%) of the 19 urgent alerts came through during work hours and were managed by the Department of Psychiatry’s care coordinator. The 2 emergent alerts were managed by the VBHC, neither of which required an emergency room referral or in-person well-being check.

### Postintervention Survey: Patients and Providers

Of the 87 participants who completed the postintervention feasibility and satisfaction survey, the median score for ease of starting the system, ease of use, and surveys being delivered at convenient times were 9, 10, and 9, respectively, on a scale of 0 (strongly disagree) to 10 (strongly agree). Median score for participant satisfaction with the Rhythms system within their MHC app was 8.

Themes from the free text indicated that respondents most liked that Rhythms gave them a greater awareness of their own mental health status, the feeling of being taken care of by their provider, and its ease of use. The most common complaint about Rhythms (mentioned by n=18, 21%) was having to keep the app open. Only 3 (3%) reported concerns about being “tracked.”

When providers were surveyed about their experience with Rhythms, they noted that the platform provided them with information they would not otherwise have had and information that was directly actionable. For example, 1 provider commented that “...the sleep section was the most useful for me to review. I am very surprised that one of my patients is only averaging about 5 hours of sleep per night, so that is definitely something we will talk about.” Another commented that “The more frequent assessment was great. I don't think patients remember their condition for more than a few days. I was able to reach out to a couple of patients based on worsening in [their] data.”

### Qualitative Interviews: Patients

Qualitative interviews with 19 (22%) of the 87 completers revealed that all but 2 were well established in the CU and UCH system for their mental health care, having received care there for at least 3 years. Most agreed to participate in the study because they felt positively about research (n=10, 11%), thought it would be easy (n=4, 5%), or thought it would be helpful to either themselves (n=3, 3%) or others (n=3, 3%). Setup and initial use of Rhythms was reported by 13 (68%) of those interviewed as being “simple, intuitive, and unobtrusive,” although 5 (26%) reported some technical challenges, including difficulty with changing settings, granting permissions, or finding specific features. With respect to completing the self-report measures and their frequency, 63% (n=12) of respondents reported completing them at the time of the prompt. In general, respondents were okay with the frequency of measures (n=3, 3%) or agreed that weekly (n=3, 3%) or every 2 weeks (n=4, 5%) would be acceptable, although a few (n=2, 2%) preferred monthly or felt surveys were sent too often (n=1, 1%).

Overall, interview participants reported positive perceptions of Rhythms, finding it easy to use (n=8, 9%) and enjoying its use (n=5, 6%). With respect to what was liked most about Rhythms, there was consensus that the platform promoted greater provider connection (n=4, 5%) and provider awareness of patients’ health status (n=6, 7%), contributing to a feeling of being “looked out for” (n=4, 5%). Participants also reported improved self-awareness of one’s own mental health status (n=5, 6%). What was liked least about Rhythms was having to keep the app open (n=7, 8%), with related concern for battery life (n=4, 5%); the wording of a response message to completed surveys notifying patients that they might be contacted based on their responses (n=6, 7%); concerns about what would happen if answers to questions were negative (n=3, 3%); not receiving immediate feedback from Rhythms; and accuracy of the data if the person did not have their phone with them or were traveling outside of Colorado.

Roughly half of those interviewed endorsed having had a helpful discussion about their Rhythms data with their provider, while others indicated that their provider did not mention their data during their appointment, or they did not have an appointment during the 6 weeks of tracking. The most common recommendation for system improvement was the ability to access and track their own data in a visual format, an improvement that is now available to users. Illustrative quotations about positive aspects of Rhythms were as follows:

…I had one two-week period where it was like, everything’s catastrophic…which solicited the call from [my provider], through the patient portal. “So I saw your responses. What’s, the scoop?”…I liked the fact that somebody was looking out for me. It just—I’m, I’m an introvert. I don't want to bother people. You know, am I really so bad that I need to interrupt somebody today? I don’t want to be a bother. But I was able to experience the effectiveness of the system, because [my provider] reached out to me. So, from a patient point of view, I can tell you, that particular item worked.

And then there was one survey when I was starting to feel a little more anxious than I normally do. I got asked to complete a survey. And I did. And I know when I completed that survey, [my provider] reached out to me and said, “Hey, I noticed that you're feeling a little more anxious than you seem to be normally.” So, I was. I’m not sure what prompted that. But I thought it was interesting that at a time I kind of had a need to communicate with [my provider], the system seemed to have recognized that.

Overall, I think that it is a wonderful tool to have in one’s toolbox to help support mental wellness.

Illustrative quotations regarding concern about Rhythms were as follows:

Also, I remember first time and a few times after that, too, I got - because I know sometimes when you answer things in a way that is concerning, they say someone will call after every survey, I got that message. Sometimes it was someone may call and sometimes someone will call…I remember because one time I left my phone at home and I went out and I was like, oh my god, someone’s gonna call and they’re gonna message. Yeah, that message was a little scary sometimes…

I didn’t like hearing that I may or may not receive a call from a provider, I would have liked it if it was more definitive.

Like, I don’t sleep with my mobile device…and I don’t always exercise with my phone.

### Reliability

The Rhythms system performed well during the pilot study with no outages and no major bugs detected.

## Discussion

### Principal Findings

Here, we describe the use, including acceptability, of the smartphone platform Rhythms by patients and providers in an academic department of psychiatry. Overall, the system integrated well with the health system’s EHR and clinical workflow and was able to be deployed with very few technical issues to a group of 104 participants. The algorithm for urgent and emergent alerts allowed the team to identify concerning changes in patient mental health status and may be considered by other clinical groups wishing to use Rhythms. The lack of adverse outcomes during this period suggests that the algorithm was set conservatively enough to capture those who are in need of outreach, but not so sensitive as to lead to inappropriate alerts that could unnecessarily burden the patient or overwhelm the monitoring team.

An impressive percentage (63/87, 72%) of study participants remained on the platform and continued to use it without any compensation beyond the end of the 6-week study, suggesting they found the app helpful. This is in distinction to the findings from Kopka and colleagues [11] showing that use of 4 out of 10 popular mental health apps dropped below 20% within the 2 weeks of initiation. None of the 10 apps chosen by participants in Kopka and colleagues [11] collected passively acquired data or were integrated into the individual’s health record. In our study, poststudy interviews revealed that passively collected data lead to a greater connection with one’s provider and was a major strength of Rhythms. Participants also provided important feedback regarding the functionality (ie, ability to track ratings visually over time and receiving immediate feedback) of Rhythms that has now been incorporated into the platform. Importantly, privacy was not a major concern expressed by participants in this study, unlike those in some [26-29] but not all previous studies [12].

The concern about having to leave the app open is real, but this did not deter the majority of individuals from remaining on the app. While providers reported that Rhythms is a useful tool in their clinical practice, some participants reported that their provider did not discuss their Rhythms data during their appointment. This may reflect the timing of Rhythms use and the availability of collected data in relation to participants’ appointments, or it may be indicative of differing levels of provider adoption of the technology and how they incorporated it into their own practices. The use of objective measurements for patient care, which is quite common in most fields of medicine, is only recently making its way into the practice of psychiatry. We are encouraged that when the Rhythms data were brought into the session, the patients reported a benefit. While scalable technology holds great promise for improving psychiatric treatment, the acceptance and use of technology in psychiatry would require a culture change if measurement assisted care is to become a reality. Since the COVID-19 pandemic, many psychiatrists and other mental health specialists are now comfortable using videoconferencing for patient visits. However, incorporating data collected from apps and wearables is an unfamiliar step in the therapeutic interaction.

Likewise, many health systems have not yet embraced linkage of apps used to collect PGHD for mental health care purposes with their EHR. When there is collection of these data through medical records standards initiative such as Fast Healthcare Interoperability Resources and other platforms [30], information is not easily collated across multiple patient-derived sources. Recent advances have been made in this area through platforms, such as mindLAMP from the Digital Psychiatry Program at Beth Israel Deaconess Medical Center of Harvard Medical School, which has the power to incorporate PGHD collected by multiple apps and wearables and offers tools to help configure data such that they are more readily usable for research or clinical care [31]. Within this context, the benefit of Rhythms is that it can be seamlessly integrated into the patient’s EHR such that the provider can view passively acquired data as well as ratings with 1 click in the patient’s chart. The alert algorithm is strong and can help guide others in their use of Rhythms to detect emerging patient crises.

Those psychiatrists who incorporated Rhythms data into their clinical care reported that it improved their understanding of their patients’ clinical status and that they used these data in their treatment planning. Likewise, patients appreciated the added connection to their provider, and there were no reports of increased provider burden across the study.

Several limitations should be considered. In total, 45.2% (178/394) of potential participants did not respond to either a general inquiry sent through MHC or an email letter sent by their provider. It is unclear why they did not respond to messages through the EHR, although previous research suggests that patient preferences for communication method are influenced by the type of information to be received [32]. Willingness to participate in a study that requires downloading an app onto one’s personal phone may require direct interaction with one’s provider and a clear discussion of the potential benefits for their treatment. The sample predominantly identified as women and White, limiting generalizability of these findings to other populations. The relatively short duration of this initial trial (6 weeks for each participant) and the relatively small number of patient and provider participants was also a limitation. Though the clinical value of Rhythms was quickly apparent, a longer period of monitoring is needed to determine the economic impact of remote patient monitoring of mental health status on health care use and costs. All study participants were currently in active mental health treatment and, on the whole, relatively stable. How Rhythms would perform in a more acutely ill population or those with psychosis within the greater health system is unknown but should be considered in future studies, as psychiatric disorders can negatively impact one’s motivation and ability to engage with an app and their overall health care [33,34].

In summary, Rhythms is a user-friendly, digital platform that can be embedded into a health system’s EHR and used to identify patients who are experiencing a worsening of their mental health between visits with their mental health care provider. The AI algorithms are based upon passively acquired data, limiting the need for patient surveys except when deviations in data suggest a deterioration in the patient’s clinical status. The culture surrounding the use of technology as part of measurement assisted mental health care will become more welcoming as studies such as ours show the ease and clinical benefits of using remote patient monitoring tools.

## Abbreviations

AI: artificial intelligence
ASRM: Altman Self-Rating Mania Scale
C-SSRS: Columbia-Suicide Symptom Severity Rating Scale
COMIRB: University of Colorado Multiple Institution Review Board
CU: University of Colorado
EHR: electronic health record
GAD-7: Generalized Anxiety Disorder 7-Item
HIPAA: Health Insurance Portability and Accountability Act
MDD: major depressive disorder
MHC: MyHealthConnection
PCP: primary care physician
PGDH: patient-generated health data
PHQ-9: Patient Health Questionnaire 9-Item
SDK: software development kit
UCH: University of Colorado Hospital
UCHealth: University of Colorado Health
VBHC: Virtual Behavioral Health Center

## Data Availability

The datasets generated and analyzed during this study are available from the corresponding author on reasonable request.
